# Regulation of ERα-dependent breast cancer metastasis by a miR-29a signaling

**DOI:** 10.1186/s13046-023-02665-6

**Published:** 2023-04-20

**Authors:** Jinhui Lü, Qian Zhao, Yuefan Guo, Danni Li, Heying Xie, Cuicui Liu, Xin Hu, Suling Liu, Zhaoyuan Hou, Xunbin wei, Deyou Zheng, Richard G. Pestell, Zuoren Yu

**Affiliations:** 1 Key Laboratory of Arrhythmias of the Ministry of Education of China, Research Center for Translational Medicine, Shanghai East Hospital, Tongji University School of Medicine, 150 Jimo Road, Shanghai, 200120 China; 2 Jinzhou Medical University, Liaoning, China; 3 Fudan University Shanghai Cancer Center, Shanghai Cancer Hospital, Shanghai, 201321 China; 4 Shanghai Jiao Tong University, Shanghai, 200240 China; 5 Departments of Genetics, Neurology, and Neuroscience, Albert Einstein College of Medicine, 1300 Morris Park Avenue, Bronx, NY 10462 USA; 6 Pennsylvania Cancer and Regenerative Medicine Research Center, and Baruch S. Blumberg Institute, 3805 Old Easton Road, Doylestown, PA 18902 USA; 7 The Wistar Cancer Center, Philadelphia, PA 19107 USA

**Keywords:** ERα, miR-29a, Breast cancer, Metastasis

## Abstract

Malignant breast cancer (BC) remains incurable mainly due to the cancer cell metastasis, which is mostly related to the status of Estrogen receptor alpha (ERα). However, our understanding of the mechanisms through which ERα regulates cancer cell metastasis remains limited. Here we identified a miR-29a-PTEN-AKT axis as a downstream signaling pathway of ERα governing breast cancer progression and metastasis. Two estrogen response element (ERE) half sites were identified in the promoter and enhancer regions of miR-29a, which mediated transcriptional regulation of miR-29a by ERα. Low level of miR-29a showed association with reduced metastasis and better survival in ERα+ luminal subtype of BC. In contrast, high level of miR-29a was detected in ERα- triple negative breast cancer (TNBC) in association with distant metastasis and poor survival. miR-29a overexpression in BC tumors increased the number of circulating tumor cells and promoted lung metastasis in mice. Targeted knockdown of miR-29a in TNBC cells in vitro or administration of a nanotechnology-based anti-miR-29a delivery in TNBC tumor-bearing mice in vivo suppressed cellular invasion, EMT and lung metastasis. PTEN was identified as a direct target of miR-29a, inducing EMT and metastasis via AKT signaling. A small molecular inhibitor of AKT attenuated miR-29a-induced EMT. These findings demonstrate a novel mechanism responsible for ERα-regulated breast cancer metastasis, and reveal the combination of ERα status and miR-29a levels as a new risk indicator in BC.

**Supplementary Information:**

The online version contains supplementary material available at 10.1186/s13046-023-02665-6.

Estrogen receptor alpha (ERα) is a nuclear receptor expressed in many cell types in both normal and disease states [1]. In mammary epithelia, upon activation by its ligand estrogen, ERα interacts with co-transcriptional factors FOXA1 and GATA3 to maintain luminal lineage and promote cell proliferation [2]. In human breast cancer, ~ 50–70% of patients have high expressions of both estrogen receptor and progesterone receptor, representing a hormone-dependent cancer type, called luminal subtype [3]. Moreover, single-nucleotide polymorphisms (SNPs) have been frequently reported in ERα gene, raising risks to develop breast cancer. As such, ERα status has been considered as an important prognostic indicator in breast cancer. It is widely used for determining breast cancer subtypes and selecting therapy methods [4]. Luminal breast cancer is characterized by positive ERα status, high differentiation, reduced metastasis, and response to estrogen therapy, resulting in prolonged survival. In contrast, basal-like breast cancer including triple negative breast cancer (TNBC) does not express ERα, and the patients are characterized by poor prognosis, frequent metastasis and poor survival.

Relapse and distant metastasis in ~ 20–30% of patients with breast cancer occur after a few months or even decades after treatment. Metastatic breast cancer is largely incurable, and frequently leads to death [5]. There is an urgent need to understand the mechanisms regulating breast cancer metastasis, and thereby develop novel therapeutic approaches. For patients with the TNBC subtype, which accounts for ~ 15–20% of breast cancer, a high prevalence of metastasis and poor survival correlate with the lack of hormone receptors ERα, progesterone receptor (PR) and HER2 [6, 7]. ERα inhibits EMT-related transcriptional factors in breast cancer, including Slug [8] and Snail [9]. Although the association between metastasis frequency and ERα status in breast cancer has been well defined, the mechanisms by which estrogen-ERα signaling controls the cancer cell metastasis is poorly understood.

MiRNAs are a class of small non-coding RNAs with 19–25 nucleotides in length, playing important roles in control of gene expression at the post-transcriptional level [10]. The aberrant expression of miRNAs in breast cancer is well documented [11]. A group of miRNAs are involved in the regulation of metastatic breast cancer. Notably, the miRNA expression profiling is closely related to the tumor features [12]. The different expression patterns of miRNAs in ERα + and ERα- human breast cancer cells and tumors have been frequently reported [13–15], including downregulation of miR-17/20, miR-145 and let-7 in ERα + breast cancer [16–18] and upregulation of miR-221/222 in ERα- breast cancer [19]. ERα interacts with miRNAs. ERα mRNA is a direct target of miR-206, miR-130, miR-145, et al. [17, 20, 21]. On the other hand, ERα regulates the expression of several miRNAs at the transcriptional level. ERα represses the expression of miR-221/222 through interacting with its promoter by recruiting the co-repressors NCoR and SMRT [22]. A negative feedback loop between miR-206 and ERα-signaling was reported in luminal A subtype of breast cancer [23, 24]. However, the mechanisms by which ERα-signaling governs miRNAs function in breast cancer metastasis remains to be fully elucidated.

Herein, we found a significant association between the upregulation in miR-29a and the induction of EMT in ERα- breast cancer. High level of miR-29a was significantly correlated with poor survival in metastatic breast cancer. Knockdown of miR-29a in TNBC cells suppressed EMT in vitro and inhibited tumor metastasis in vivo. miR-29a overexpression increased the number of circulating tumor cells, and promoted tumor metastasis to the lung. ERα was identified as a transcriptional regulator of miR-29a in breast cancer, responsible for its downregulation in luminal subtype and upregulation in TNBC subtype. miR-29a-PTEN-AKT was demonstrated to be a downstream signaling of ERα, determining the breast cancer progression and metastasis. Application of a nanotechnology-based miR-29a inhibitor showed anti-metastasis efficacy in the tumor-burden mice with breast cancer. The current study is the first to demonstrate a mechanism through which ERα-miR-29a signaling controls metastasis in breast cancer.

## Methods and materials

### Human breast tumor samples

Human breast tumor samples were collected from Tongji University Shanghai East Hospital and Fudan University Shanghai Cancer Center. All the procedures were approved by the Institutional Review Board (IRB) of Shanghai East Hospital (#2019TJDX107). All patients were provided with written informed consent form.

### Animals

All procedures on the animal studies were approved by the Institutional Animal Care and Use Committee of the Tongji University School of Medicine. 6–8-week-old female BALB/c nude mice were purchased from Shanghai Silaike Laboratory Animal Co. Ltd (Shanghai, China).

### Cell lines and cell culture

All breast cancer cell lines used in this study were originally purchased from American Type Culture Collection (ATCC), and maintained in our lab. The culture condition includes 37 °C, 5% CO_2_ and Dulbecco's Modified Eagle's Medium (DMEM) medium supplemented with 10% fetal bovine serum (FBS) and 1% penicillin–streptomycin.

### Vectors

The vectors expressing either pre-miR-29a or control in lentiviral vector pCDH-CMV-MCS-EF1-copGFP were purchased from System Biosciences (Mountain View, CA). Packaging plasmids psPAX2 and pMD2.G were used for the lentiviral transduction. Cells stably overexpressing miR-29a were purified by fluorescence-activated cell sorting (FACS). The pcDNA3.1 plasmid was used to overexpress ERα and Pten. pBABE-IRES-GFP retroviral vector encoding human gene v-Src was used to transduce MCF-10A cells as previously described [25, 26].

### Oligos and transfection

The sequences of gene-specific oligos are as follows: si- ERα sense: 5’-UGAGUAACAAAUUCAUGGAGdTdT-3’, si-negative control (NC) sense: 5’- CUCCAUGCCUUUGUUACUCAdTdT-3’, miR-29a mimic sense: 5’- UAGCACCAUCUGAAAUCGGUUAdTdT-3’, miR-NC sense: 5’-UGGGCGUAUAGACGUGUUACACdTdT-3’, anti-miR-29a: 5’ -UAACCGAUUUCAGAUGGUGCUA-3’. anti-miR-NC: 5’-GUGUAACACGUCUAUACGCCCA-3’. All oligos were synthesized by GenScript (Nanjing, China). Oligos were transiently transfected into cells using RNAiMAX (Invitrogen) following the manufacturer’s instruction with a final concentration of 30 nM.

### miRNA Screening and Real-Time PCR Analysis

Trizol reagent (Invitrogen) was used to extract total RNA. First-strand complementary DNA (cDNA) of miRNAs was synthesized from total RNA with the M&G miRNA Reverse Transcription kit (miRGenes, Shanghai, China) in accordance with the manufacturer’s instruction. All of the primer oligos were synthesized by GenScript (Nanjing China). Quantitative real time PCR assays were carried out using SYBR Green Master Mix (Applied Biosystem, Life Technologies) on the ABI 7900 HT Sequence Detection System (Applied Biosystem, Life Technologies).

### Wound-healing assay

Cells were seeded into 12-well culture plates to achieve 90 to 95% confluence. A vertical wound was created in each well using a 10 μl pipette tip. The cells were cultured in DMEM medium with reduced serum (0.1% FBS). Images were captured in 0 h, 24 h and 48 h to assess the closure rate of the wound.

### Cell invasion assay

Transwell chambers (8 μm pores, Corning, USA) were pre-coated with the ECM Gel (E1270, Sigma-Aldrich, USA). 2 × 10^4^ cells were seeded in the upper chambers with serum-free medium, and normal medium in the bottom chambers. After 12 h of incubation, cells adherent to the lower surface of the chambers were stained with 0.4% crystal violet for photographing and quantitative analysis.

### Western blot

The regular procedure for western blot analysis was described previously [27]. Primary antibodies were diluted with 1:1,000, including Fibronectin (sc-8422, Santa Cruz), Snail1 (sc-271977, Santa Cruz), PTEN (sc-7974, Santa Cruz), ERα (sc-787, Santa Cruz), Vimentin (sc-32322, Santa Cruz), Akt (4691, Cell Signaling Technology), p-Akt (4060 T, Cell Signaling Technology), p65 (8242, Cell Signaling Technology), p-P65 (3033 T, Cell Signaling Technology), β-Catenin (8480 T, Cell Signaling Technology), α-Tubulin (ab-7291, Abcam), β-Actin (sc-47778, Santa Cruz) and GAPDH (sc-47724, Santa Cruz). HRP-linked anti-rabbit IgG (7074S, Cell Signaling Technology) and HRP-linked anti-mouse IgG (7076S, Cell Signaling Technology) were used as secondary antibodies with a dilution of 1:5,000.

### In vivo flow cytometry (IVFC)

Circulating tumor cells in mice were tracked in vivo through IVFC as we described previously [27]. Briefly, the tumor-burden mouse was anesthetized and placed on the flow cytometry platform. The major arteries in the mouse ear were visualized under illumination. An artery with a diameter of 50 µm was chosen for data acquisition. The fluorescence signal in cells would be excited when passing through the laser slit. The emitted fluorescence was collected by a photomultiplier tube (PMT) and digitized with a data acquisition card at a sampling frequency of 5 kHz. Each mouse was detected for continuous 30 or 60 min each time as indicated.

### Chromatin immunoprecipitation

MCF-7 cells were cross-linked with formaldehyde. Chromatin immunoprecipitation was performed as described previously [28] using ERα antibody (13258S; Cell Signaling Technology). PCR was performed using the immunoprecipitated DNA as template. The primer sequences designed in the promoter and enhancer regions of miR-29a were provided in Supplemental Table S1.

### Luciferase reporter assay

pMIR-REPORT luciferase reporter vectors carrying either WT PTEN 3′ UTR or MU PTEN 3′ UTR (point mutation to the two binding sites of miR-29a) were applied to determine the direct interaction between miR-29a and PTEN mRNA. Cells were seeded in 24-well plates at a density of 5 × 10^4^ cells/well. After cell adherence, 1.0 μg of PTEN 3′ UTR firefly reporter and 0.2 μg of renilla vector were co-transfected using Lipofectamine 2000 (Invitrogen). Luciferase activities were measured using the Dual-luciferase reporter assay system (Promega, Madison, WI, USA) by AutoLumat in 18–24 h after transfection.

### In vivo tumor xenograft model

5 × 10^5^ of breast cancer cells per mouse were mixed with matrigel and injected into the fat pat of the fourth mammary gland of female nude mice to grow mammary tumors. The IVFC analysis and tumor metastatic analysis were performed on these mice. For the cancer treatment, Zn_0.4_Fe_2.6_O_4_@SiO_2_ magnetic nanoparticles carrying either anti-miR-29a inhibitor or negative control (1 mg/kg body weight per dose) were tail-vein injected to the tumor-burden mice at the indicated timepoints. Immediately after each injection, a piece of magnet was placed close to the tumor for 1 h to help the nanoparticles enriched in the tumor tissues. The volume of tumors was measured every 3 days until day 30 after cell transplantation when all the mice were sacrificed. Tumors were separated, weighted and applied for further analysis. Lung metastasis was examined.

### Public database

The TCGA database (https://www.xiantao.love/products) was used to analyze the gene expression levels and survival.

### Statistical analysis

Data are presented as mean ± SEM unless stated otherwise. The standard two-tailed student’s t-test and one-way ANOVA followed by Least-Significant Difference (LSD) were used in statistical comparisons. *p* < 0.05 was considered as statistical significance.

## Results

### Negative correlation between the miR-29a expression level and ERα status in human breast cancer

**Fig. 1:**
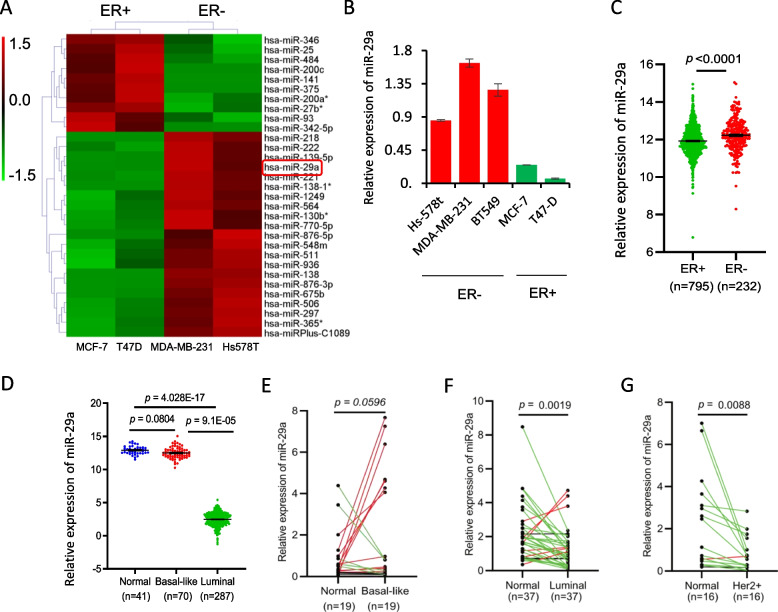
Negative correlation between miR-29a and ERα in expression in human breast cancer. **A** Heatmap of differentially expressed miRNAs in ERα + (MCF-7 and T47D) and ERα- (MDA-MB-231 and Hs578T) human breast cancer cell lines. **B** Validation of aberrant expression of miR-29a in 3 ERα- and 2 ERα + breast cancer cell lines. Data are presented as the mean ± SEM (*n* = 3). **C** Relative expression levels of miR-29a in 795 ERα + and 232 ERα- breast cancer patients from TCGA database. **D** Relative expression levels of miR-29a in luminal and basal-like subtypes of breast cancer from the TCGA database. **E** Upregulation of miR-29a in 19 TNBC breast cancer tumors in comparison to the matched adjacent tissues. **F** Downregulation of miR-29a in 37 luminal breast cancer tumors in comparison to the matched adjacent tissues. **G** Downregulation of miR-29a in 16 HER2 + breast cancer tumors in comparison to the matched adjacent tissues

In order to determine the molecular differences between ERα + and ERα- breast cancer, we performed a miRNA screening analysis in ERα + (MCF-7 and T47D) and ERα- (MDA-MB-231 and Hs578T) human breast cancer cell lines. A subset of differentially expressed miRNAs were identified, including miR-29a and miR-221/222 (Fig. 1A). miR-221/222 has been well studied by our previous work [29]. Herein, we confirmed the aberrant expression of miR-29a. A negative correlation between the miR-29a level and ERα status was observed in ERα-based human breast cancer cell lines (Fig. 1B) and cancer tumors (Fig. 1C). miR-29a showed high levels in TNBC and low levels in the luminal subtype of breast cancer (Fig. 1D-G).

### ERα repressed miR-29a expression at the transcriptional level

**Fig. 2:**
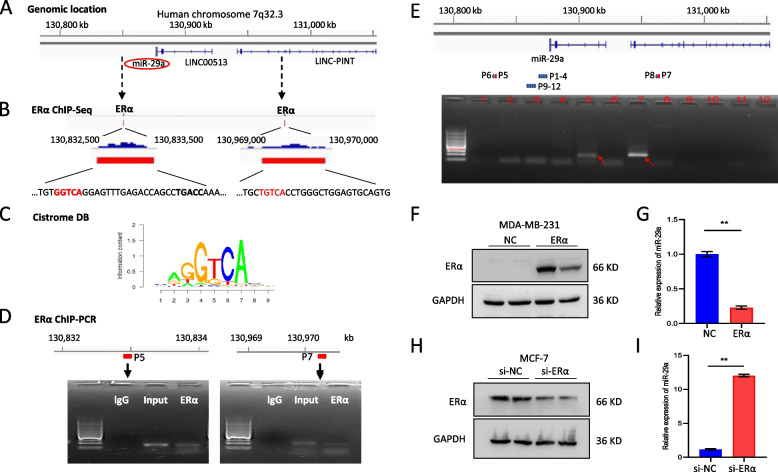
ERα repressed miR-29a expression at the transcriptional level. **A** Genome region of miR-29a at Chromosome 7q32.3. **B** ChIP-seq identified two ERE half sites in the promoter region and enhancer region of miR-29a. **C** Prediction of a conserved ERα binding motif -G/TGTCA- by CistromeDB Toolkit. **D** ChIP-PCR using MCF-7 cells to validate the two ERE half sites to the genome of miR-29a indicated in (**B**). IgG was used as a negative control, input was used for positive control. **E** Additional screening of ERα-binding sequences at the genome region of miR-29a by applying 12 pairs of primers to run PCR, followed by agarose gel electrophoresis (lanes 1–12). Primer pair 5 (P5) represented the predicted binding site at the enhancer region, and primer pair 7 (P7) represented the predicted binding site at the promoter region were indicated with red arrows. **F**, **G** Overexpression of ERα in MDA-MB-231 cells (**F**) suppressed the expression of miR-29a (**G**). **H**, **I** Knocked down of ERα in MCF-7 cells (**H**) promoted the expression of miR-29a (**I**). Data are presented as the mean ± SEM (*n* = 3). **p < 0.01

In order to determine the mechanism by which ERα regulates miR-29a, we screened DNA sequence in the human miR-29a genome region at Chromosome 7q32.3 (Fig. 2A). Based on an ERα ChIP-seq dataset, two ERα-binding peaks were identified in the miR-29a promoter and enhancer region, respectively (Fig. 2B), carrying a conserved ERE half-sites motif -GGTCA- or -TGTCA- (Fig. 2C). In order to validate this binding interaction, we conducted chromatin immunoprecipitation assay in MCF-7 cells using an ERα antibody, followed by PCR analysis using 12 pairs of primers (Supplemental Table S1) (Fig. 2D and E). As a result, the primer pair #7 representing a promoter binding fragment and the primer pair #5 representing an enhancer binding fragment obtained amplification from the ERα antibody-immunoprecipitated genomic DNA, but not from the IgG control (Fig. 2D and E). In addition, we overexpressed ERα in MDA-MB-231 breast cancer cells (ERα-) or knocked down ERα in MCF-7 cells (ERα +), respectively, followed by quantitative analysis of miR-29a. As shown in Fig. 2F-I, ERα negatively regulated the expression of miR-29a in breast cancer cells.

### miR-29a induced EMT and promoted cell migration and invasion in breast cancer cells in vitro

**Fig. 3:**
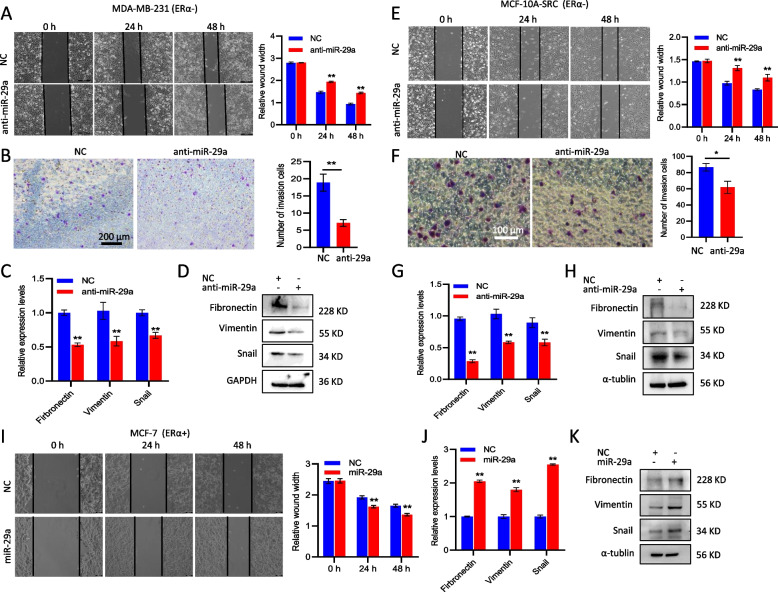
miR-29a induced EMT and promoted cell migration and invasion. **A**, **B** Wound healing assay (**A**) and Transwell assay (**B**) indicated suppression of cell migration and invasion in MDA-MB-231 cells after knockdown of miR-29a. **C**, **D** Reduced expression of EMT marker genes (vimentin, fibronectin and snail) at both mRNA (**C**) and protein (**D**) levels in MDA-MB-231 cells after knockdown of miR-29a. **E**–**H** Similar assays to A-D, cell migration, invasion and EMT analyses in MCF-10A-Src cells with or without knockdown of miR-29a. **I** Wound healing assay indicated induction of cell migration in the miR-29a-overexpressing MCF-7 cells. **J**, **K** Overexpression of miR-29a in MCF-7 cells significantly promoted the expression of EMT marker genes (vimentin, fibronectin and snail) at both mRNA (**J**) and protein (**K**) levels. Data are presented as the mean ± SEM (*n* = 3). *p < 0.05, **p < 0.01

In order to determine the function of miR-29a in breast cancer, we firstly knocked down miR-29a in two ERα- cell lines MDA-MB-231 and MCF-10A-Src (MCF-10A cells transformed by v-Src, Supplemental Fig. S1), followed by cellular function assays. As seen in Fig. 3A and B, knockdown of miR-29a in MDA-MB-231 cells significantly suppressed cellular migration and invasion. EMT marker genes including vimentin, fibronectin and snail decreased in expression at both mRNA and protein levels after knockdown of miR-29a (Fig. 3C-D). Similar results were observed in MCF-10A-Src cells (Fig. 3E-H). In contrast, miR-29a overexpression promoted cell migration, invasion, and induced EMT in MDA-MB-231 cells (Supplemental Fig. S2) and MCF-10A-Src cells (Supplemental Fig. S3).

In addition, an ERα + breast cancer cell line MCF-7 was analyzed for miR-29a function. Enforced expression of miR-29a in MCF-7 cells (Supplemental Fig. S4) significantly increased cellular migration (Fig. 3I) and induced EMT (Fig. 3J-K).

### miR-29a increased circulating tumor cells (CTC) and promoted tumor metastasis to the lung in vivo

**Fig. 4:**
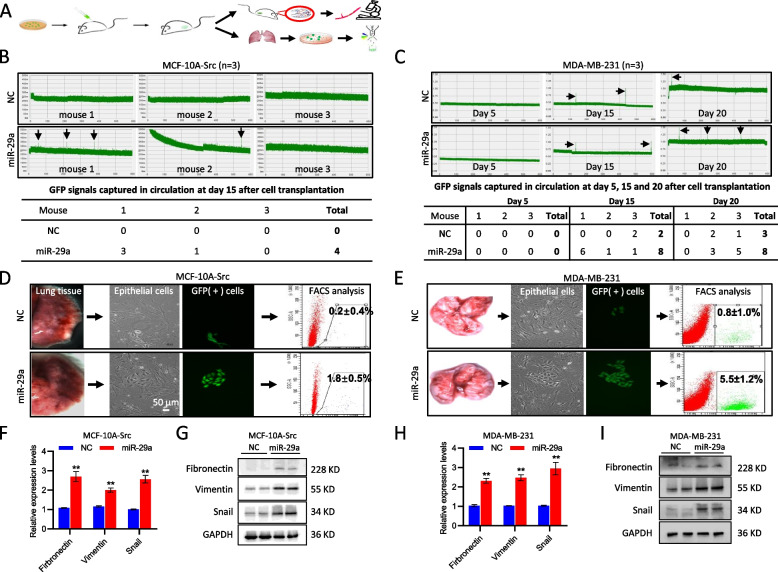
miR-29a promoted tumor metastasis to the lung in vivo. **A** Schematic representation of the procedure for analyzing circulating tumor cells (CTCs) and lung metastasis in mice. **B** Real-time tracking of CTCs by capturing the GFP fluorescence signals in the mammary tumor mice at day 15 after transplantation with MCF-10A-Src cell with or without overexpressing miR-29a. In vivo flow cytometry (IVFC) assay was applied. *n* = 3 in each group. Representative CTC signals were indicated with arrows. **C** Real-time tracking of CTCs in the mammary tumor mice at day 5, day 15 and day 20 after transplantation with MDA-MB-231 cell with or without overexpressing miR-29a. *n* = 3 in each group. Representative CTC signals were indicated with arrows. **D**, **E** Single cell suspension was prepared from the lung tissues of mammary tumor mice derived from either MCF-10A-Src (**D**) or MDA-MB-231 (**E**) cell transplantation. FACS analysis demonstrated promoted tumor cell metastasis to the lung by overexpression of miR-29a. **F**-**I** Overexpression of miR-29a induced the expression of EMT markers vimentin, fibronectin and snail at both mRNA and protein levels in the mammary tumors derived from either MCF-10A-Src (**F**, **G**) or MDA-MB-231 (**H**, **I**) cell transplantation. Data are presented as the mean ± SEM (*n* = 3). **p < 0.01

In order to determine the function of miR-29a in regulating breast cancer in vivo, we stably overexpressed miR-29a in MDA-MB-231 or MCF-10A-Src cells, labeled with GFP, and transplanted into immunodeficient nude mice, followed by analyses of mammary tumor growth, CTC in blood and metastasis in the lung (Fig. 4A). miR-29a overexpression did not affect primary tumor growth (Supplemental Fig. S5). CTCs were real-time tracked through capturing the GFP fluorescence signals by applying in vivo flow cytometry technology. We first performed the CTC analysis in the tumor mice at day 15 after MCF-10A-Src cell transplantation. 4 CTC signals were captured in total within 60 min in three miR-29a mice, compared to 0 signal in three controls (Fig. 4B). Then a more aggressive TNBC cell line MDA-MB-231 was applied for further validation. CTCs were analyzed in the mice at day 5, day 15 and day 20 after cell transplantation, respectively. The results show a greater number of CTCs in the miR-29a-overexpressing mice than controls (Fig. 4C).

In association with the mammary tumor growth, CTCs seed distant organs, leading to metastasis. After euthanasia of the mammary tumor-burden mice derived by transplantation of breast cancer cells with or without overexpression of miR-29a, lung tissues were assessed into single cell suspension. The GFP positive metastatic cancer cells in the lung were observed under fluorescence microscopy, and quantified by FACS analysis using flow cytometry. As shown in Fig. 4D and E, overexpression of miR-29a promoted MCF-10A-Src cells to metastasize to the lung from 0.2% to 1.8% of the cell percentage (Fig. 4D), and from 0.8% to 5.5% for MDA-MB-231 cells as well (Fig. 4E).

We further examined the EMT regulation by miR-29a in vivo. Consistent with the in vitro results, miR-29a induced the mRNA and protein levels of the EMT markers vimentin, fibronectin and snail in the mammary tumors (Fig. 4F-I).

### PTEN-AKT signaling mediated the miR-29a-induced breast cancer metastasis

**Fig. 5:**
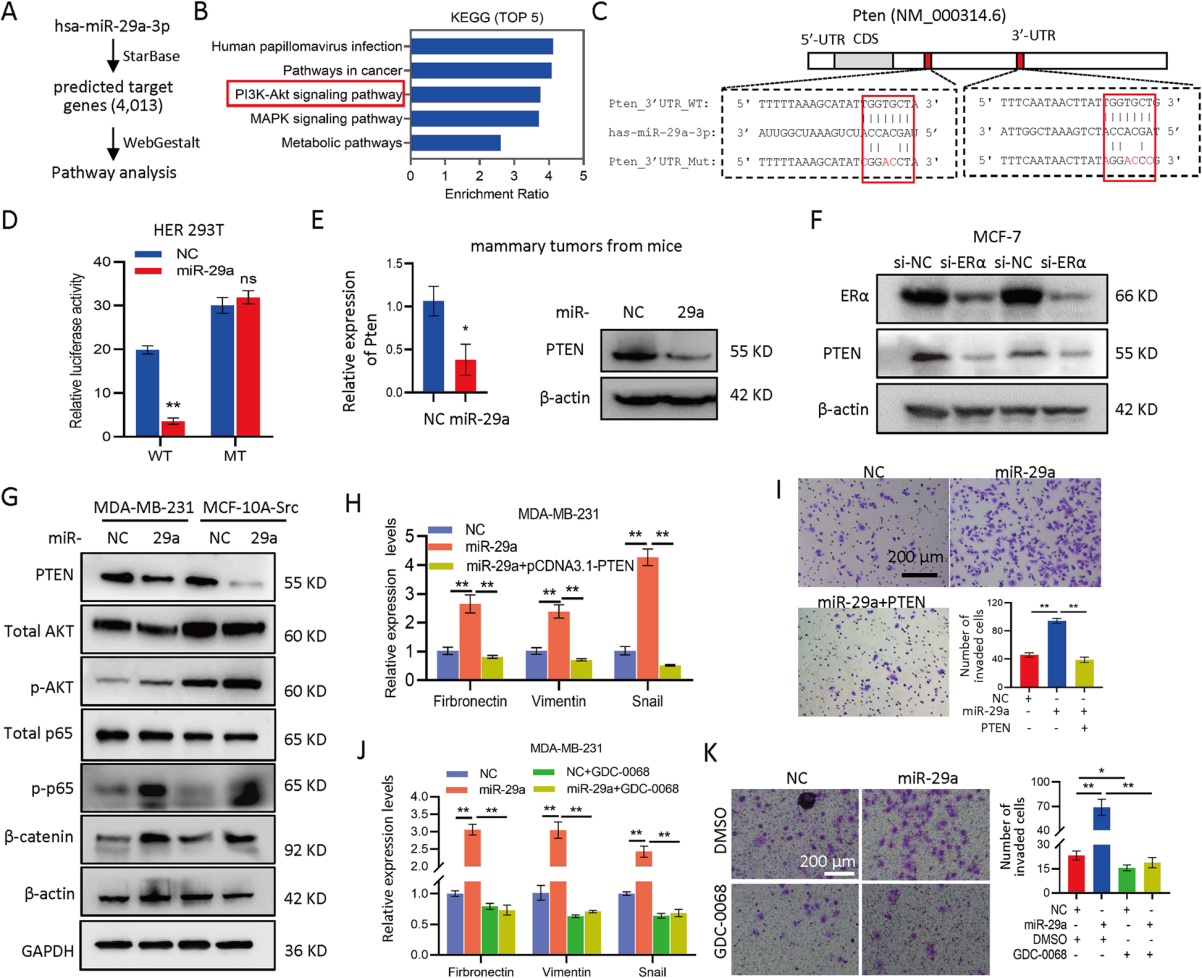
PTEN-AKT signaling mediated the miR-29a-induced breast cancer metastasis. **A** Procedure for miR-29a target gene prediction and pathway analysis. **B** Top 5 pathways from KEGG analysis of the 4,013 predicted target genes of miR-29a. **C** Sequence alignment between PTEN 3’UTR and miR-29a. Wild type (WT) or point mutated (MT) PTEN 3′UTR were cloned into pGL-3 Luciferase reporter vector. **D** Luciferase reporter assay demonstrated inhibition of 3′UTR of WT PTEN by miR-29a, but not for mutated PTEN. **E** miR-29a suppressed the expression of PTEN at both mRNA and protein levels in the mammary tumors derived from MCF-10A-Src. **F** Western blot indicated suppression of PTEN in MCF-7 cells by knocking down of ERα. **G** Western blot indicated suppression of PTEN and induction of p-AKT, p-p65 and β-catenin by miR-29a overexpression in both MCF-10A-Src and MDA-MB-231 cells. **H** miR-29a induced the expression of EMT markers fibronectin, vimentin and snail in MDA-MB-231 cells, which was attenuated by transfection with pcDNA3.1-Pten. **I** miR-29a induced cell invasion, which was attenuated by transfection with pcDNA3.1-Pten in in MDA-MB-231 cells. **J**, **K** Application of a AKT inhibitor GDC-0068 attenuated the miR-29a-induced EMT gene expression (**J**) and miR-29a-induced cell invasion (**K**) in MDA-MB-231 cells. Data are presented as the mean ± SEM (*n* = 3). *p < 0.05, **p < 0.01, ns means non-significance

In order to determine the molecular mechanism through which miR-29a induced EMT and metastasis in breast cancer, we predicted the potential target genes of miR-29a (Fig. 5A), and applied for KEGG pathway analysis. PI3K-AKT and MAPK were two of the top five pathways with gene enrichment (Fig. 5B). PTEN has a well-defined function in human cancer inhibiting PI3K-AKT signaling. Two binding sites of miR-29a were identified in the 3’UTR of PTEN (Fig. 5C). Luciferase reporters carrying either wide type (WT) or mutated (MT) 3’UTR of PTEN demonstrated a direct interaction between miR-29a and PTEN through the two binding sites (Fig. 5C and D). miR-29a suppressed the expression of PTEN in breast cancer cells (Fig. 5E). We knocked down ERα in MCF-7 cells, resulting in induction of miR-29a and suppression of PTEN (Fig. 5F). Analysis on thousands of breast cancer tumor samples indicated higher level of PTEN in ERα + breast cancer than that in ERα- breast cancer (Supplemental Fig. S6). These results further confirmed suppression of miR-29a-PTEN signaling by ERα in breast cancer.

In addition to PTEN, downstream genes of PI3K-AKT pathway including p-AKT, p-p65, β-catenin were examined in both MCF-10A-Src and MDA-MB-231 cells with or without overexpression of miR-29a. The results demonstrated the activation of PI3K-AKT signaling by miR-29a in breast cancer (Fig. 5G). Transcription factor p65, also known as nuclear factor NF-kappa-B p65 subunit, was activated by miR-29a overexpression. Accordingly those EMT marker gene expressions and the cell invasion were induced, which were attenuated by the introduction back of PTEN (Fig. 5H, I). Moreover, we applied GDC-0068, an AKT inhibitor, to the miR-29a-overexpressing MDA-MB-231 cells, leading to the rescue of EMT and cell invasion (Fig. 5J, K). These results further validated the EMT induction by the miR-29a-Pten-AKT axis in human breast cancer.

### Clinical relevance of miR-29a in breast cancer patients

**Fig. 6:**
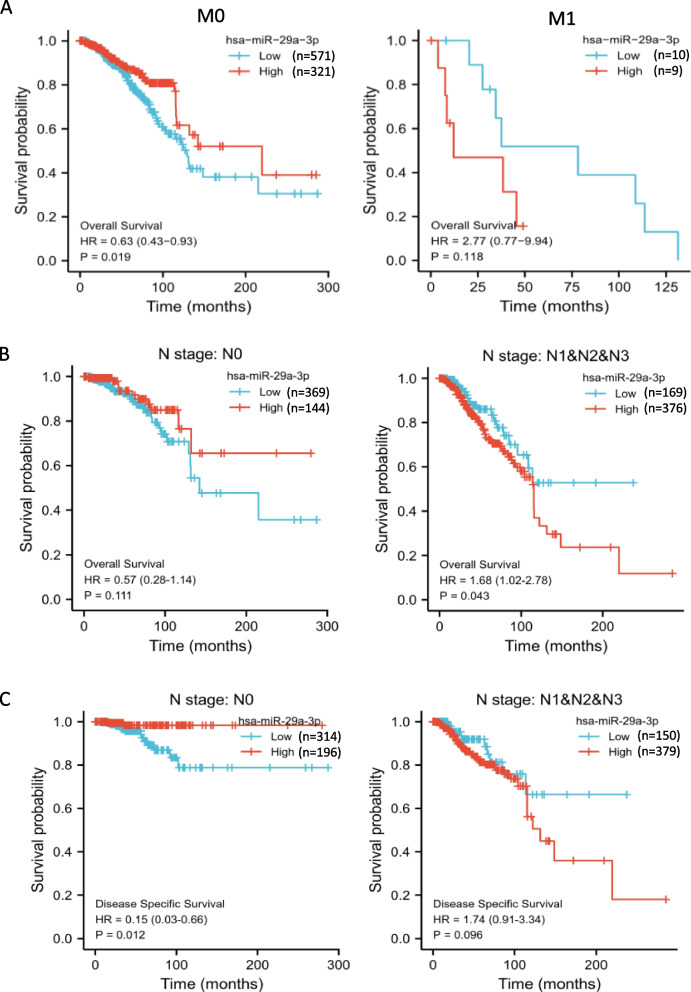
Clinical relevance of miR-29a in breast cancer patients. **A**, **B** Negative correlations between the overall survival rate and the miR-29a level in breast cancer patients with either distant metastasis (M1 stage) (**A**) or lymph node metastasis (N1-N3) (**B**). **C** A negative correlation between the disease specific survival and the miR-29a level in breast cancer patients with lymph node metastasis (N1-N3). In contrast, breast cancer patients without metastasis (M0 or N0 stages) showed positive correlations between the survival rate and miR-29a expression. The TCGA database was used for analysis

In order to further reveal the clinical relevance between miR-29a, PTEN and ERα status and TNM stage in breast cancer, breast cancer patients in the TCGA database were analyzed. As shown in Supplemental Fig. S7, an increasing trend of miR-29a in expression in ERα- breast cancer patients while decreasing trend in ERα + patients were observed from early stage to late stage (Supplemental Fig. S7A). PTEN showed the opposite trends with miR-29a (Supplemental Fig. S7B). Moreover, a negative correlation between the overall survival rate and the miR-29a level was observed in breast cancer patients with either distant metastasis (M1 stage) (Fig. 6A) or lymph node metastasis (N1-N3) (Fig. 6B), so did disease specific survival (Fig. 6C).

### Application of a miR-29a inhibitor to treat a mouse model with metastatic breast cancer

**Fig. 7:**
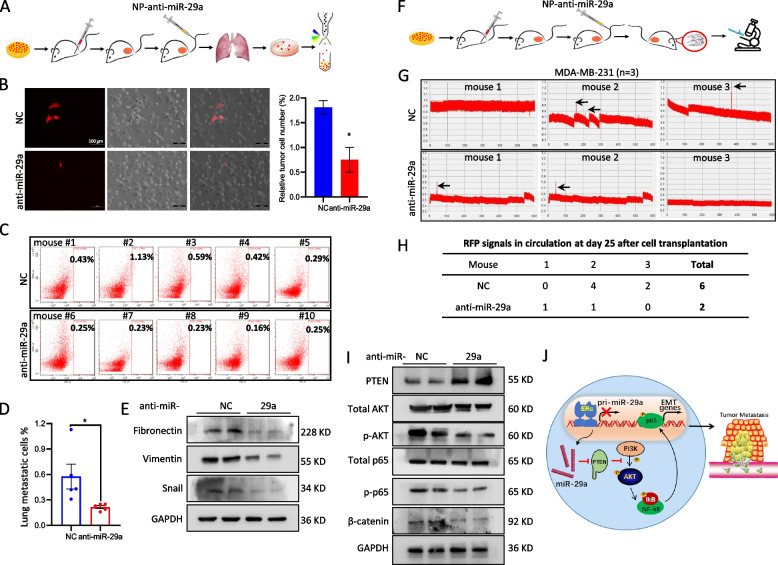
Therapeutic effect of a miR-29a inhibitor in treatment of metastatic breast cancer. **A** Schematic representation of the procedure to treat the mammary tumor mice with anti-miR-29a, followed by analysis of metastasis in the lung. **B** Representative images of the metastatic breast tumor cells (RFP-labeled MDA-MB-231) in the lung after adhesion to culturing plate from lung single cell suspension. Application of anti-miR-29a inhibitor significantly decreased the tumor metastasis (*n* = 5 in each group). **C** FACS analysis indicated the percentage of the metastatic tumor cells in the lung of each mouse (*n* = 5 in each group). **D** Quantitative analysis of **C**. Data are presented as the mean ± SEM (*n* = 5). *p < 0.05. **E** Western blot demonstrated suppression of EMT markers vimentin, fibronectin and snail in expression in the mammary tumors after treatment with anti-miR-29a inhibitor. **F** Schematic representation of the procedure to treat the mammary tumor mice with anti-miR-29a, followed by analysis of CTCs in blood. **G**, **H** Real-time tracking of CTCs in the mammary tumor mice at day 25 after transplantation with MDA-MB-231 cells with or without treatment with anti-miR-29a inhibitor. In vivo flow cytometry (IVFC) assay was applied. *n* = 3 in each group. **I** Western blot demonstrated inactivation of PI3K-AKT signaling in the mammary tumors after treatment with anti-miR-29a inhibitor, in which p-AKT, p-p65 and β-catenin showed decrease while PTEN showed increase in expression. **J** Schematic representation of the mechanism through which ERα-miR-29a signaling controls metastasis in breast cancer

In order to investigate clinical translational potential of these findings, we prepared hyaluronic acid (HA)-modified nanoparticles carrying antisense oligos (ASO) of miR-29a, and deployed these particles to treat metastatic mammary tumor mice. CTCs in the blood and lung metastasis were detected in the mice with or without treatment with anti-miR-29a (Fig. 7). As shown in Fig. 7A-E, inhibition of miR-29a in the mammary tumor mice significantly suppressed the lung metastasis, reduced the expression of EMT markers fibronectin, vimentin and snail, but did not affect the primary tumor growth (Supplemental Fig. S8). The number of CTCs in circulation decreased from 6 in NC group (*n* = 3) to 2 in miR-29a inhibitor group (*n* = 3) (Fig. 7F-H). Western blot analysis further confirmed upregulation of PTEN and inactivation of AKT signaling in the tumor mice upon administration with miR-29a inhibitor (Fig. 7I).

## Discussion

ERα, as a receptor of estrogen, plays important roles in the development, progression and treatment of human breast cancer. ERα status is closely correlated with tumor grade, distant metastasis and prognostic outcome in breast cancer. For example, ERα + luminal subtype of breast cancer retains certain degree of epithelial characteristics and displays low tumor grade, weak invasiveness and rare metastasis. In contrast, ERα- TNBC subtype is associated with high invasiveness, frequent metastasis and poor prognosis [30]. In addition, acquisition of ligand‐independent ERα mutations during aromatase inhibitor therapy in breast cancer was proved to be a common mechanism of hormonal therapy resistance [31]. As such, ERα signaling is widely believed to help maintaining epithelial phenotypes by suppressing EMT [32]. However, metastasis still occurs frequently in breast cancer, even in a subpopulation of ERα + patients*.* It is an urgent need to determine the key mechanism regulating ERα-dependent metastasis in breast cancer, which will shed light on identification of novel therapeutic targets.

ERα signaling governs multiple pathways that regulate breast cancer progression and metastasis. Breast cancer tumor progression in vivo is promoted by NF-kappa-B [33, 34]. NF-kB inhibition by ERα signaling led to suppression of tumor metastasis in breast cancer [35]. In addition, a set of EMT-related transcriptional factors and non-coding RNAs showed regulation by ERα [36]. Herein, we demonstrated a novel regulatory network between ERα, miR-29a and PTEN-AKT signaling to control tumor metastasis in human breast cancer, in which ERα negatively regulates the expression of miR-29a at the transcriptional level. miR-29a targets PTEN, thereby activates PI3K-AKT signaling to trigger EMT and metastasis (Fig. 7J). Our findings can well explain the low level of miR-29a, high level of PTEN and rare metastasis in ERα + luminal breast cancer subtype, while the high level of miR-29a, low level of PTEN and frequent metastasis in ERα- TNBC breast cancer.

MiR-29a has been reported to regulate pulmonary fibrosis, hepatic fibrosis and aneurysm formation through targeting extracellular matrix components [37, 38]. Moreover, aberrant expression of miR-29a has been found in multiple cancer types [39]. Function of miR-29a in regulation of human cancer seems controversial and complex in literature [40]. miR-29a may function as a tumor suppressor or an oncogene depending on tumor type, tumor grade, or even tumor stage. For example, downregulation of miR-29a was reported in tumor tissues from glioma [41], lung [42, 43], cervix [44], bladder [45], prostate [46], stomach [47] and colon [48], functioning as a tumor suppressor to inhibit cancer cell proliferation and metastasis. Upregulation of miR-29a was reported in nodular cholangiocarcinoma [49]. In breast cancer, miR-29a showed upregulation to promote oncogenesis [50]. While another study reported miR-29a to induce the cell cycle arrest at G0/G1 phase in certain types of breast cancer [51]. Our current study determined the expression pattern of miR-29a in subtype-dependent and stage-dependent manners in breast cancer. In luminal subtype, miR-29a was suppressed by ERα, functioning as a tumor suppressor to inhibit EMT and metastasis. In TNBC, especially at late stages, miR-29a showed induction in expression, facilitating distant metastasis.

In order to determine the therapeutic potential of targeting miR-29a or AKT signaling in treatment of breast cancer, a kind of magnetic nanoparticles carrying miR-29a inhibitor was applied to treat the TNBC tumor-burden mice. In vivo flow cytometry analysis of CTCs and fluorescence activated cell sorting of metastatic tumor cells in the lungs clearly demonstrated suppression of cancer metastasis by targeting miR-29a. In addition, application of a small molecular inhibitor of AKT showed inhibition of tumor cell invasion in TNBC. In conclusion, the current study not only reveals a miRNA signaling-based mechanism mediating ERα-controlled metastasis in human breast cancer, but also provides a novel therapeutic strategy to treat patients with metastatic breast cancer.

## Conclusions

Herein we found a novel miR-29a-PTEN-AKT axis to mediate ERα-controlled breast cancer progression and metastasis. Targeted knockdown of miR-29a in breast cancer cells in vitro or targeted delivery of nanotechnology-based miR-29a inhibitor into mammary tumor-bearing mice in vivo suppressed cellular invasion, reduced the number of circulating tumor cells, and inhibited distant metastasis. In addition, administration of a small molecular inhibitor of AKT attenuated miR-29a-induecd EMT. The current study demonstrates the theragnostic value of combining ERα status with miR-29a levels in patient for determining therapeutic strategy and predicting prognosis in breast cancer.

## Supplementary Information

**Additional file 1: Table S1. **Sequence of 12 primer pairs in the genome region of miR-29a.

**Additional file 2: Figure S1.** Quantitative validation of miR-29a knockdown in MDA-MB-231 (A) and MCF-10A-SRC (B) cells. **Figure S2.** Overexpression of miR-29a promoted cell migration and invasion, and induced EMT in MDA-MB-231 cells. **Figure S3.** Overexpression of miR-29a promoted cell migration and invasion, and induced EMT in MCF-10A-SRC cells. **Figure S4.** Quantitative validation of miR-29a overexpression in MCF-7 cells. **Figure S5.** Image and weight of the mammary tumors derived from mice transplanted with MCF-10A-SRC cells with or without overexpression of miR-29a. **Figure S6.** Higher levels of PTEN in ERα+ breast cancer tumors (n=808) than that in ERα- ones (n=238). **Figure S7.** Correlations between miR-29a, PTEN and ERα in different stages of breast cancer patients. **Figure S8.** Image and weight of the mammary tumors from the mice treated with miR-29a inhibitor or control.

**Additional file 3. **

## Data Availability

All data are included in this published article and supplementary files. All materials we generated by ourselves are shareable upon request.

## Abbreviations

BC: Breast cancer
TNBC: Triple negative breast cancer
EMT: Epithelial-mesenchymal transition
cDNA: Complementary DNA
miRNA: MicroRNA
ATCC: American Type Culture Collection
FBS: Fetal bovine serum
CMV: Cytomegalovirus
FACS: Fluorescence-activated cell sorting
IVFC: In vivo flow cytometry
ChIP-Seq: Chromatin immunoprecipitation sequencing
PCR: Polymerase Chain Reaction
ASO: Antisense oligonucleotide
CTC: Circulating tumor cells
NC: Negative control
ERα: Estrogen receptor alpha
PR: Progesterone receptor
TGF-β: Transforming Growth Factor-β
PI3K: Phosphatidylinositide 3-kinases
