# Supplementary material for: Regulation of ERα-dependent breast cancer metastasis by a miR-29a signaling
Source: J Exp Clin Cancer Res. 2023 Apr 20;42:93. doi: 10.1186/s13046-023-02665-6 (PMC10116798; doi:10.1186/s13046-023-02665-6)

REGULATION OF ERA-DEPENDENT BREAST CANCER METASTASIS BY A MIR-29A SIGNALING

Jinhui Lü[1†], Qian Zhao[1†], Yuefan Guo[1], Danni Li[1], Heying Xie[1,2], Cuicui Liu[3], Xin Hu[3], Suling Liu[3], Zhaoyuan Hou[4], Xunbin wei[4], Deyou Zheng[5], Richard G. Pestell[6,7] and Zuoren Yu[1*]

Supplemental Figure Legends

Figure S1: Quantitative validation of miR-29a knockdown in MDA-MB-231 (A) and MCF-10A-SRC (B) cells. Data are presented as the mean ± SEM (n=3). **p<0.01.

Figure S2: Overexpression of miR-29a promoted cell migration and invasion, and induced EMT in MDA-MB-231 cells. A: Quantitative validation of miR-29a overexpression in MDA-MB-231 cells. B, C: Wound healing assay (B) and Transwell assay (C) indicated promotion of cell migration and invasion in miR-29a-overexpressing MDA-MB-231 cells. D: Induced expression of EMT marker genes vimentin, fibronectin and snail in miR-29a-overexpressing MDA-MB-231 cells. Data are presented as the mean ± SEM (n=3). **p<0.01.

Figure S3: Overexpression of miR-29a promoted cell migration and invasion, and induced EMT in MCF-10A-SRC cells. A: Quantitative validation of miR-29a overexpression in MCF-10A-SRC cells. B, C: Wound healing assay (B) and Transwell assay (C) indicated promotion of cell migration and invasion in miR-29a-overexpressing MCF-10A-SRC cells. D: Induced expression of EMT marker genes vimentin, fibronectin and snail in miR-29a-overexpressing MCF-10A-SRC cells. Data are presented as the mean ± SEM (n=3). **p<0.01.

Figure S4: Quantitative validation of miR-29a overexpression in MCF-7 cells. Data are presented as the mean ± SEM (n=3). **p<0.01.

Figure S5: Image and weight of the mammary tumors derived from mice transplanted with MCF-10A-SRC cells with or without overexpression of miR-29a.

Figure S6: Higher levels of pten in ERα+ breast cancer tumors (n=808) than that in ERα- ones (n=238).

Figure S7: Correlations between miR-29a, pten and ERα in different stages of breast cancer patients. A: Increasing trend of miR-29a in expression in ERα- breast cancer patients while decreasing trend in ERα+ patients from early stage to late stage. B: Pten showed opposite trends with miR-29a.

Figure S8: Image and weight of the mammary tumors from the mice treated with miR-29a inhibitor or control.

Supplemental Figure S1

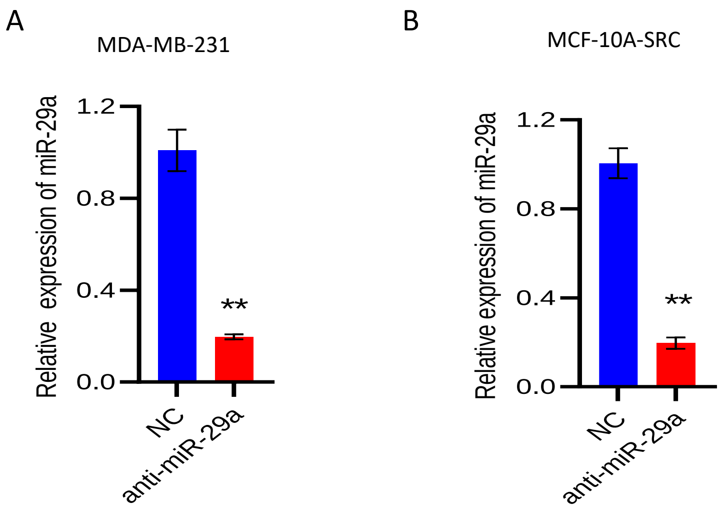

Supplemental Figure S2

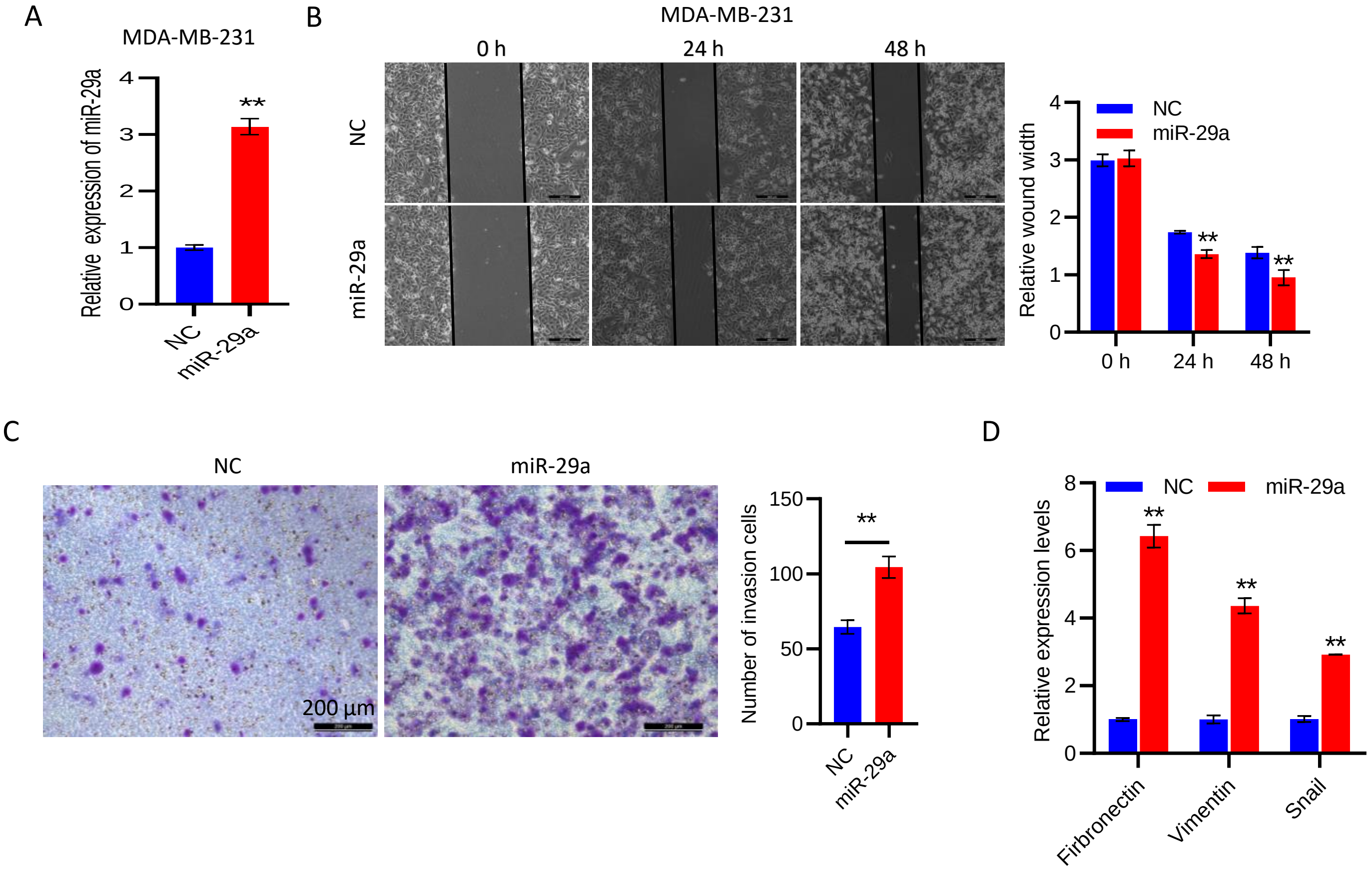

Supplemental Figure S3

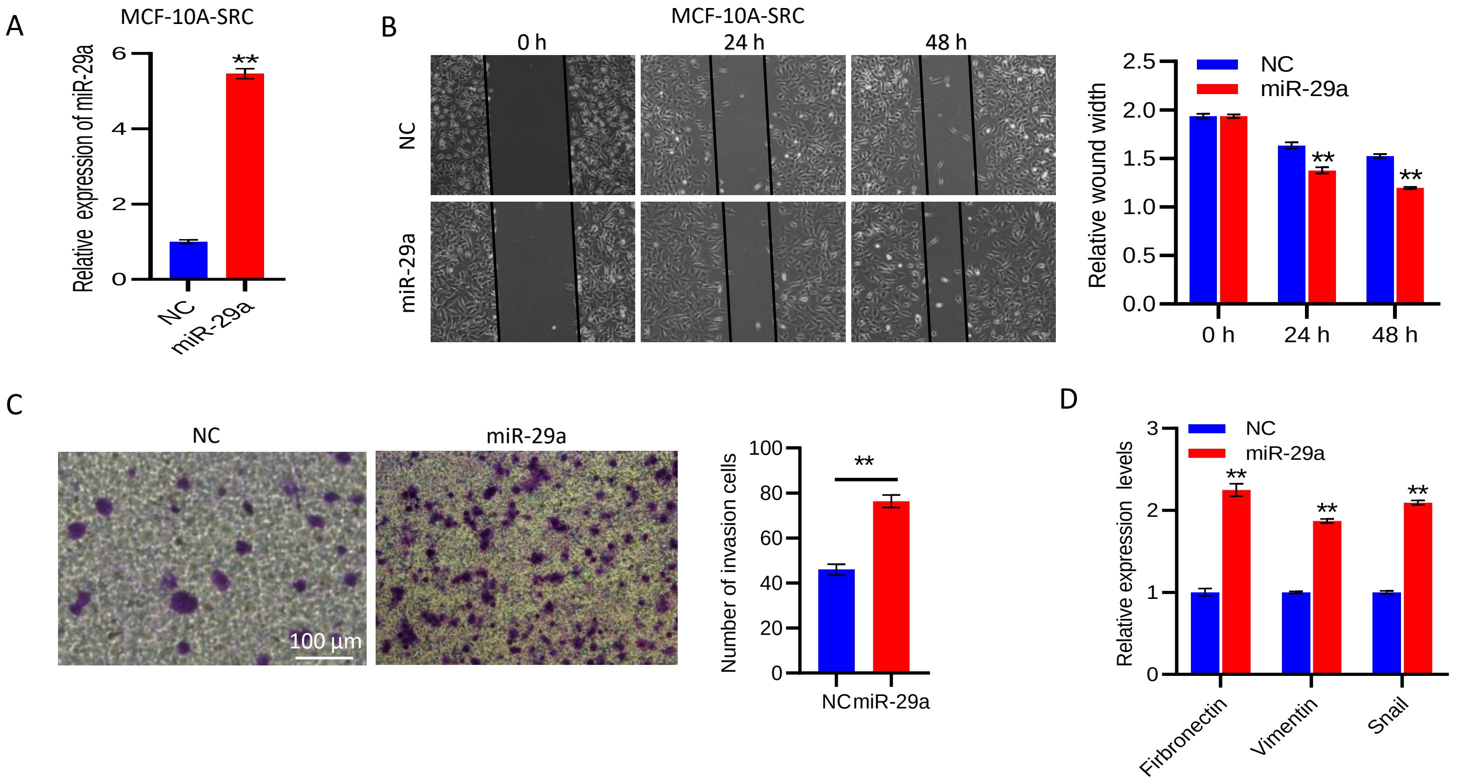

Supplemental Figure S4

A

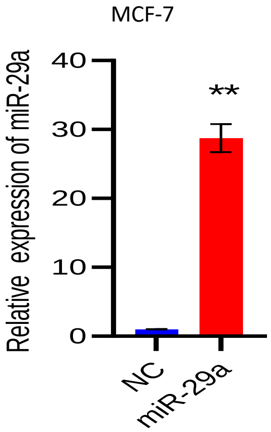

Supplemental Figure S5

A

MCF-10A-Src

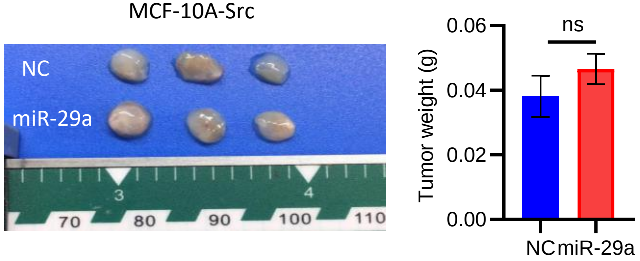

B

MDA-MB-231

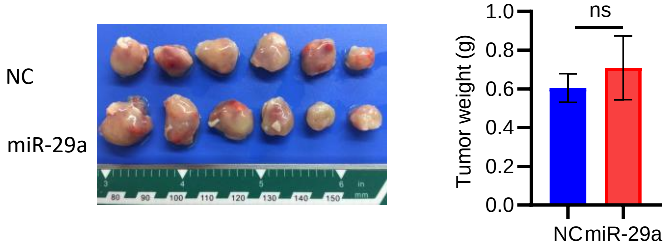

Supplemental Figure S6

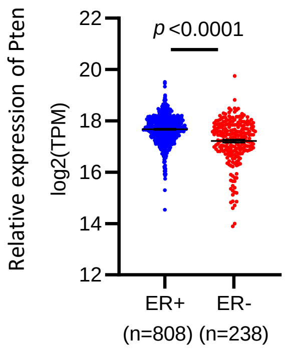

Supplemental Figure S7

A

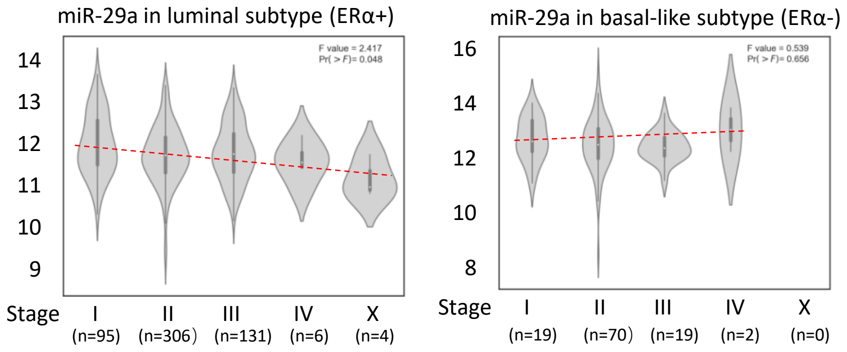

B

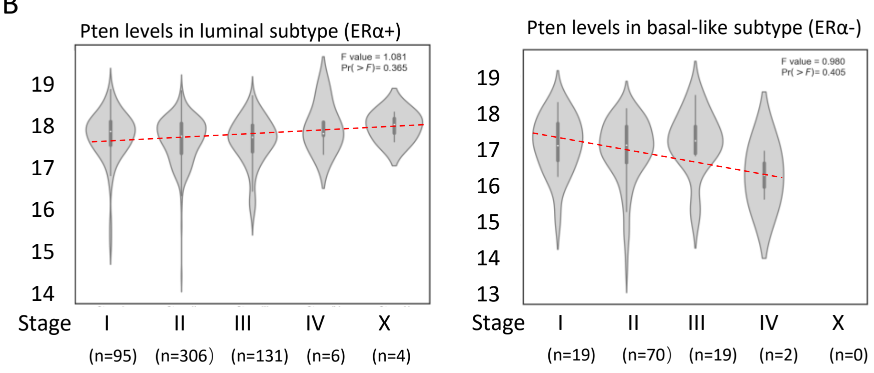

Supplemental Figure S8

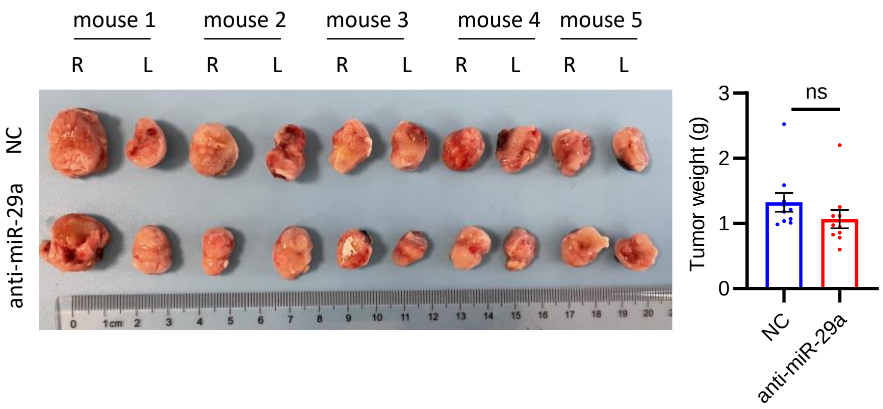

Supplement: Supplementary file 2 — Additional file 2: Figure S1. Quantitative validation of miR-29a knockdown in MDA-MB-231 (A) and MCF-10A-SRC (B) cells. Figure S2. Overexpression of miR-29a promoted cell migration and invasion, and induced EMT in MDA-MB-231 cells. Figure S3. Overexpression of miR-29a promoted cell migration and invasion, and induced EMT in MCF-10A-SRC cells. Figure S4. Quantitative validation of miR-29a overexpression in MCF-7 cells. Figure S5. Image and weight of the mammary tumors derived from mice transplanted with MCF-10A-SRC cells with or without overexpression of miR-29a. Figure S6. Higher levels of PTEN in ERα+ breast cancer tumors (n=808) than that in ERα- ones (n=238). Figure S7. Correlations between miR-29a, PTEN and ERα in different stages of breast cancer patients. Figure S8. Image and weight of the mammary tumors from the mice treated with miR-29a inhibitor or control. [file 13046_2023_2665_MOESM2_ESM.pdf]
