# Supplementary figures and images for: Regulation of ERα-dependent breast cancer metastasis by a miR-29a signaling
Source: J Exp Clin Cancer Res. 2023 Apr 20;42:93. doi: 10.1186/s13046-023-02665-6 (PMC10116798; doi:10.1186/s13046-023-02665-6)

Figure 2F

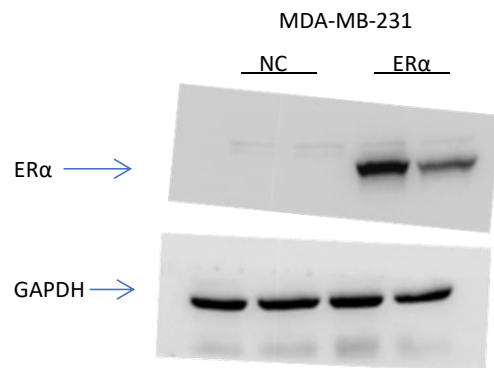

Figure 2H

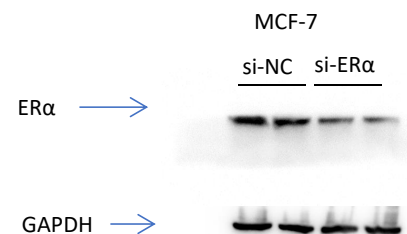

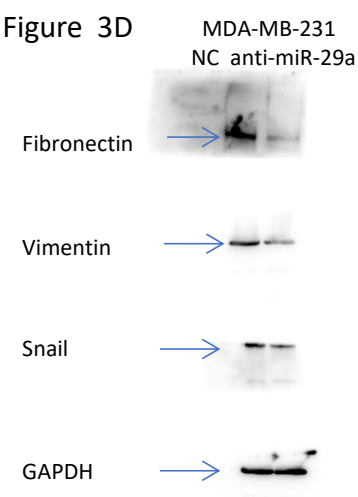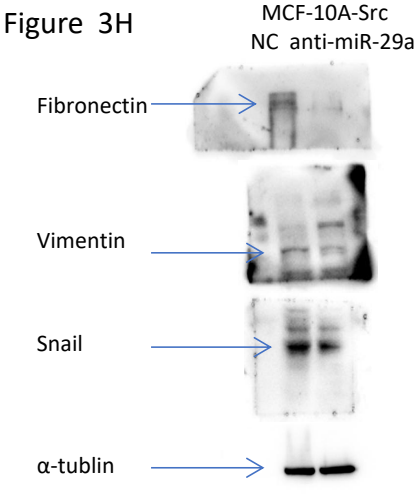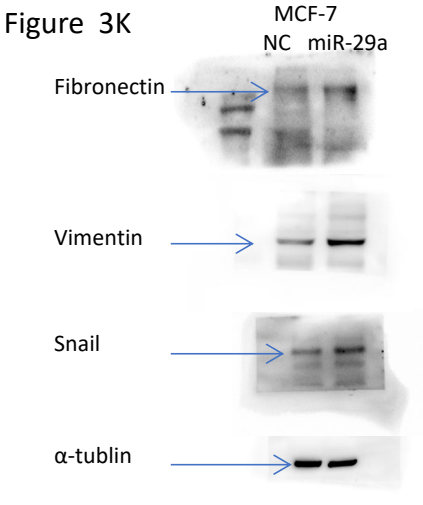

Figure 4G

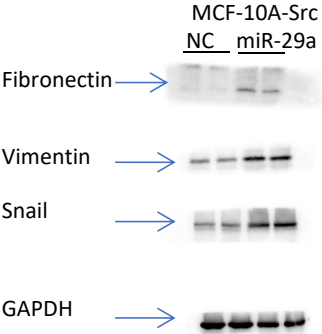

Figure 4I

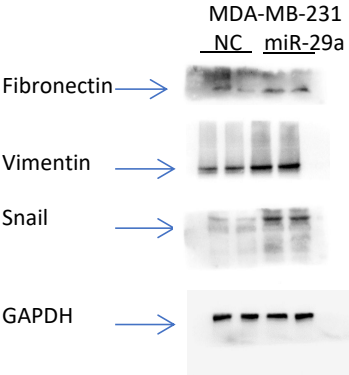

Figure 5E

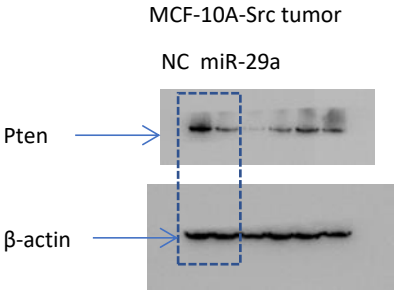

Figure 5F

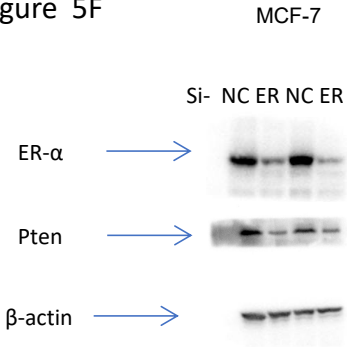

Figure 5G

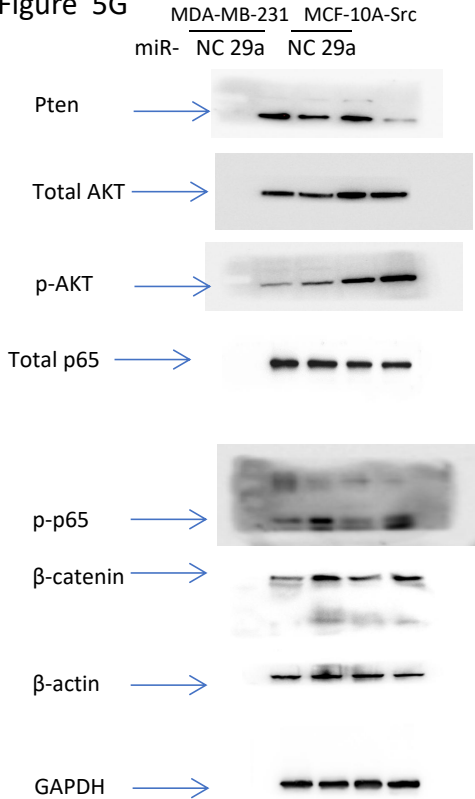

Figure 7E

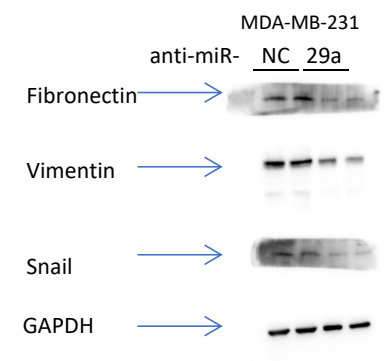

Figure 7I

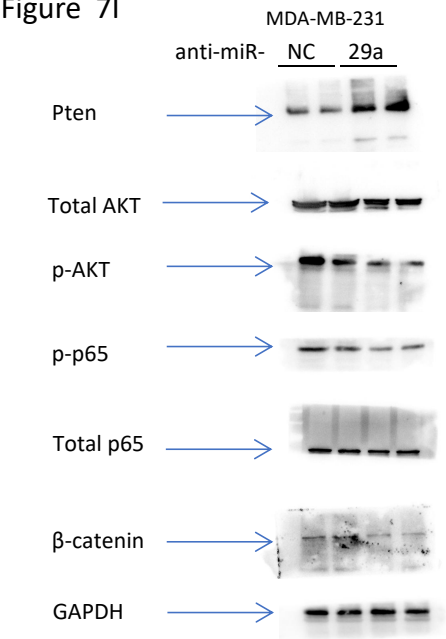

Supplement: Supplementary file 3 — Additional file 3. [file 13046_2023_2665_MOESM3_ESM.pdf]
